# COVID-19 with repeated positive test results for SARS-CoV-2 by PCR and then negative test results twice during intensive care: a case report

**DOI:** 10.1186/s13256-020-02534-2

**Published:** 2020-10-07

**Authors:** Masafumi Kanamoto, Masaru Tobe, Tomonori Takazawa, Shigeru Saito

**Affiliations:** Department of Anesthesiology and Intensive Care Unit, Gunma University Hospital, 3-39-15 Showa, Maebashi, Gunma Japan

**Keywords:** COVID-19, RT-PCR, Repeat positivity/negativity

## Abstract

**Background:**

Determining the infectiousness of patients with coronavirus disease 2019 is crucial for patient management. Medical staff usually refer to the results of reverse transcription polymerase chain reaction tests in conjunction with clinical symptoms and computed tomographic images.

**Case presentation:**

We report a case of a 62-year-old Japanese man who twice had positive and negative test results by polymerase chain reaction for severe acute respiratory syndrome coronavirus 2 over 48 days of hospitalization, including in intensive care. His respiratory symptoms and computed tomographic imaging findings consistent with coronavirus disease 2019 improved following initial intensive care, and the result of his polymerase chain reaction test became negative 3 days before discharge from the intensive care unit. However, 4 days after this first negative result, his polymerase chain reaction test result was positive again, and another 4 days later, he had a negative result once more. Eight days after the second polymerase chain reaction negative test result, the patient’s test result again became positive. Finally, his polymerase chain reaction results were negative 43 days after his first hospitalization.

**Conclusions:**

This case emphasizes the importance of repeat polymerase chain reaction testing and diagnosis based on multiple criteria, including clinical symptoms and computed tomographic imaging findings. Clinical staff should consider that a negative result by polymerase chain reaction does not necessarily certify complete coronavirus disease 2019 recovery.

## Introduction

A number of cases of “unknown viral pneumonia” related to a market in Wuhan City, Hubei Province, China, were reported in December 2019. The novel severe acute respiratory syndrome coronavirus 2 (SARS-CoV-2) was identified, causing coronavirus disease 2019 (COVID-19), which rapidly spread from China to other countries all over the world. In the absence of specific therapeutic drugs or vaccines for COVID-19, it is essential to be able to detect the disease at an early stage and immediately isolate the infected person from the healthy population. According to the latest guidelines for the Diagnosis and Treatment of Pneumonitis Caused by 2019 Novel Coronavirus (Trial Version 6) published by the Chinese government, the diagnosis of COVID-19 requires testing respiratory or blood samples by reverse transcription polymerase chain reaction (RT-PCR) or gene sequencing and is considered the key indicator for hospitalization. Chest computed tomography (CT) reveals typical radiographic features in almost all patients with COVID-19, which include ground-glass opacities, multifocal patchy consolidation, and/or interstitial changes with a peripheral distribution. We now have a great deal of experience in treating patients with frequent changes from positive to negative PCR results, then back to positive and negative again. We therefore propose that accurate diagnosis and treatment of COVID-19 requires a comprehensive assessment that includes not only PCR results but also chest CT images.

## Case presentation

**Fig. 1:**
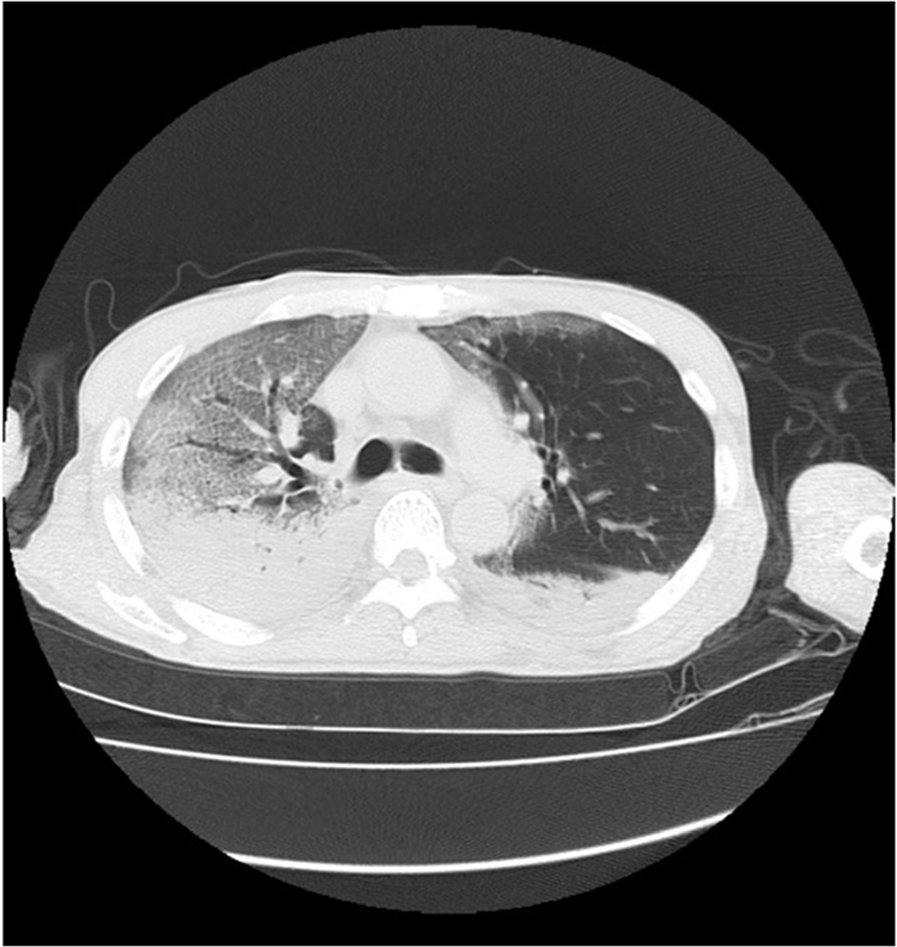
Computed tomography of the chest demonstrating bilateral patchy ground-glass opacities with interlobular septal thickening consistent with the crazy paving pattern found in patients with coronavirus disease 2019

A 62-year-old Japanese man without coexisting disease initially presented to our hospital with a persistent fever of 38.0 °C, dyspnea, and hypoxia after close contact with a coworker known to be infected with SARS-CoV-2. His oxygen saturation (SpO_2_) on room air at the time of hospitalization was 94%, and CT showed peripheral ground-glass opacities with interlobular septal thickening consistent with a “crazy paving pattern” strongly indicative of COVID-19 (Fig. 1). PCR results on the basis of a pharyngeal swab taken through the nostril were consistent with pneumonia and COVID-19. Because the patient’s SpO_2_ decreased to 88% 25 days after hospitalization despite 3 L/minute oxygen inhalation by face mask, he was transferred to the intensive care unit (ICU). He was intubated and put on a ventilator (Puritan Bennett 840, Medtronic, Tokyo, Japan; pressure control ventilation [PCV] mode, fraction of inspired oxygen [FiO_2_], 0.5, positive end-expiratory pressure [PEEP], 10 cmH_2_O; inspiratory pressure [Pi], 15 cmH_2_O; inspiratory time [Ti], 1.5 s; frequency [f], 12 per minute). Other therapeutic procedures included administration of favipiravir, and, given concerns regarding pneumonia due to other pathogens, broad-spectrum antibiotic therapy using tazobactam/piperacillin and levofloxacin was initiated. The results of blood cultures and a respiratory viral panel were negative. The patient recovered without further incident and was transferred back to a convalescence ward in an affiliated hospital after confirmation of SARS-CoV-2 negativity by PCR. However, on the same day as the transfer, the patient complained of shortness of breath and dyspnea, and his respiration rate increased to 20 breaths/minute. His SpO_2_ decreased to 86% under 10 L/minute of 100% oxygen inhalation by face mask, and he was again intubated. At this time, the finding of PCR was once again positive for SARS-CoV-2, and the patient was readmitted to our hospital and transferred back to the ICU to restart respiratory care on a ventilator (PCV, FiO_2_, 0.4; PEEP, 8 cmH_2_O; Pi, 15 cmH_2_O; Ti, 1.5 s; f, 12 per minute). Four days after readmission, his respiratory condition had improved, and his PCR results were again negative. Nine days after readmission, he was weaned off respirator care, extubated, and transferred to a COVID-19 ward in the same hospital. Over the remainder of his hospital course, the patient was treated by supportive measures and monitored for any worsening of respiratory function. Despite his respiratory condition not worsening, his PCR result again became positive 3 days after discharge from the ICU for the second time. At 8 and 11 days after this, his PCR results were negative once more. Following confirmation that his clinical condition and CT findings were stable, he was finally discharged from our hospital 54 days after his first admission.

## Discussion

For successful management of the COVID-19 pandemic, diagnosis and discharge criteria have been discussed extensively with reference to the sensitivity and specificity of the clinical and virological status of patients before discharge. The PCR test is considered the gold standard for detecting infection and is widely used for diagnosis and public heath surveillance of disease prevalence. In this report, we describe a patient who repeatedly had positive test results and then negative and positive test results again several times during the course of his COVID-19 disease. Although there are several reports of PCR reverting to positivity following a negative result, twice repeating such a positive and negative course in one patient seems to be rare. Considering safe management for clinical staff and the patient him- or herself, the importance of repeat testing and screening based on clinical symptoms and exposure history cannot be overstated. PCR has emerged as the test of choice for detection of viral nucleic acids and the infectiousness of infected individuals. Although some reports in the literature emphasize the importance of PCR screening for early containment of the disease, the sensitivity of PCR tests has been shown to be anything but perfect. A study conducted in China found that almost 25% of SARS-CoV-2-positive individuals had had a negative result in initial testing [1]. Another study reported that over 20% of infected individuals had positive test results on their third consecutive test after two initial negative results [2]. The sensitivity of PCR testing in several studies has been reported to be only 71–83%, corresponding to a false-negative rate of up to 30% [3, 4]. Considering this reported poor sensitivity of PCR for SARS-CoV-2, clinicians should be cautious when interpreting negative results of PCR testing in patients with clinical suspicion of COVID-19.

Our patient initially presented with fatigue progressing to fever, cough, and shortness of breath, symptoms that are most commonly attributable to COVID-19 pneumonia [5, 6]. However, prior to his first discharge from the ICU, the patient was completely free of these symptoms, and his PCR result was negative. Because the CT images still showed some consolidation in the right upper and middle lung lobes, we consider it possible that the virus was in fact still present and that it moved out to the pharynx during transfer to a different hospital. Although we considered that some kind of coexisting bacterial pneumonia could be the main reason for the patient’s retarded recovery as seen by CT, these CT images may in fact have more importance for evaluating COVID-19 disease. A case report stated that CT imaging should be an integral component of screening for COVID-19 in preoperative patients [7]. Typical CT findings include consolidation, vascular enhancement, air bronchus sign, and bilateral peripheral ground-glass opacities with interlobular septal thickening consistent with a “crazy paving pattern” [8]. In our patient’s case, these features were apparent at the initial admission. However, such findings were not apparent at the second admission to the ICU. These CT findings are clearly not specific for COVID-19 and may also be present in other viral or bacterial pneumonias. However, increasing numbers of clinical reports are emphasizing the efficacy of CT imaging for treating patients with this viral disease, and several clinical surveys have shown that CT imaging can enhance the accuracy of COVID-19 diagnosis over and above PCR alone [3, 9]. These recent reports support the notion that recovery from SARS-CoV-2 infection and the criteria for deciding on hospital discharge criteria should be based not only on the PCR results but also on assessment of CT images and clinical symptoms.

## Conclusions

First, a negative PCR test result confirms neither recovery from COVID-19 nor that the patient is no longer infectious. Considering the poor sensitivity of PCR, repeat testing is essential to identifying SARS-CoV-2-positive individuals at initial diagnosis. A second important conclusion is that isolation and treatment practices should be guided by a combination of testing, symptomology, and radiologic evidence at the time of discharge and should not rely solely on PCR. Careful consideration based on these multiple parameters may prevent premature discharge of the “presumed negative” patients and thus also prevent unexpected exposure of healthcare workers and the population at large.

## Data Availability

Please contact the author for data requests.
